# ﻿Morphology and multigene phylogeny revealed four new species of *Xylodon* (Schizoporaceae, Basidiomycota) from southern China

**DOI:** 10.3897/mycokeys.107.128223

**Published:** 2024-08-07

**Authors:** Qi Yuan, Changlin Zhao

**Affiliations:** 1 The Key Laboratory of Forest Resources Conservation and Utilization in the Southwest Mountains of China Ministry of Education, Key Laboratory of National Forestry and Grassland Administration on Biodiversity Conservation in Southwest China, Yunnan Provincial Key Laboratory for Conservation and Utilization of In-forest Resource, Southwest Forestry University, Kunming 650224, China Southwest Forestry University Kunming China; 2 College of Forestry, Southwest Forestry University, Kunming 650224, China Southwest Forestry University Kunming China

**Keywords:** Biodiversity, China, phylogenetic analyses, wood-inhabiting fungi, Yunnan Province

## Abstract

Fungi are one of the most diverse groups of organisms on Earth, amongst which wood-inhabiting fungi play a crucial role in ecosystem processes and functions. Four new wood-inhabiting fungi, *Xylodoncremeoparinaceus*, *X.luteodontioides*, *X.poroides* and *X.wumengshanensis* are proposed, based on morphological features and molecular evidence. *Xylodoncremeoparinaceus* is distinguished by a cream hymenial surface with a pruinose hymenophore, a monomitic hyphal system with clamped generative hyphae and ellipsoid basidiospores. *Xylodonluteodontioides* is characterised by flavescens hymenophore surface with odontioid hymenophore, monomitic hyphal system with clamped generative hyphae and ellipsoid basidiospores. *Xylodonporoides* bears coriaceous basidiomata with a poroid hymenophore surface, monomitic hyphal system with clamped generative hyphae and ellipsoid basidiospores. *Xylodonwumengshanensis* is a distinct taxon by its grandinoid hymenophore surface, monomitic hyphal system with clamped generative hyphae and ellipsoid basidiospores. Sequences of ITS and nLSU rRNA markers of the studied samples were generated and phylogenetic analyses were performed using the Maximum Likelihood, Maximum Parsimony, and Bayesian Inference methods. The phylogram, based on the ITS+nLSU rDNA gene regions, included three genera within the Schizoporaceae as *Fasciodontia*, *Lyomyces* and *Xylodon*. The four new species were grouped into the genus *Xylodon*. The topology, based on the ITS sequences, revealed that *Xylodoncremeoparinaceus* was grouped closely with *X.pruinosus*, *X.detriticus* and *X.ussuriensis*. The taxon *X.luteodontioides* was sister to *X.nesporii*. The species *X.poroides* separated from *X.pseudotropicus*, while *X.wumengshanensis* was grouped with four taxa: *X.patagonicus*, *X.radula*, *X.subtropicus* and *X.taiwanianus*.

## ﻿Introduction

Fungi are well-known as a diverse group of microorganisms that play important roles in forest ecosystems (Phookamsak et al. 2019). Wood-inhabiting fungi are essential to natural ecosystems for nutrient cycling and maintaining plant diversity (Drinkwater et al. 2017; Horwath 2017; Hyde et al. 2018; Wu et al. 2022a, b; Guan et al. 2023; Yuan et al. 2023; Deng et al. 2024a, b; Zhang et al. 2024). Schizoporaceae Jülich includes many variations of the fruiting body types amongst Hymenochaetales Oberw. (Larsson et al. 2006; Wu et al. 2022a; Guan et al. 2023; Zhang et al. 2024), in which it comprises many representative wood-inhabiting fungal taxa, including hydnoid, corticioid and polyporoid basidiomes with diverse hymenophoral and cystidial morphology (Yurchenko and Wu 2016; Riebesehl and Langer 2017; Yurchenko et al. 2017; Cui et al. 2019; Riebesehl et al. 2019; Jiang et al. 2021; Wu et al. 2022a, b; Guan et al. 2023; Deng et al. 2024a, b; Zhang et al. 2024). In addition, members of Schizoporaceae are widely distributed, causing white rot (Langer 1994; Luo et al. 2022; Guan et al. 2023; Zhang et al. 2024).

*Xylodon* (Pers.) Gray is a large genus of corticioid fungi, having a cosmopolitan distribution (Bernicchia and Gorjón 2010; Guan et al. 2023; Yurchenko et al. 2024; Zhang et al. 2024). Species of *Xylodon* inhabit dead wood of various sizes, from twigs several millimetres in diameter to large fallen trunks and cause white rot (Girometta et al. 2020; Greslebin and Rajchenberg 2000; Kotiranta and Saarenoksa 2000; Guan et al. 2023). Sometimes basidiomata of *Xylodon* species appear on living parts of trees (Yurchenko 2008) and non-woody plant remains, for example, fern rachises (Kotiranta and Saarenoksa 2000), herb stems and fallen leaves (Viner et al. 2018) and dead polypore basidiomata (Viner et al. 2023). The genus is known from almost all types of world biomes where wooden plant debris occurs, from humid to semi-arid and from seashore to the upper limit of wooden vegetation in altitudinal gradients (Yurchenko et al. 2024). This genus is typified by *X.quercinus* (Pers.) Gray (Bernicchia and Gorjón 2010) and characterised by the resupinate or effuse basidiomata with a smooth, tuberculate, grandinioid, odontioid, coralloid, irpicoid or poroid hymenophore; a monomitic or dimitic hyphal system with clamped generative hyphae; the presence of different types of cystidia; utriform or suburniform basidia; and cylindrical to ellipsoid to globose basidiospores (Gray 1821; Bernicchia and Gorjón 2010; Zhang et al. 2024). Based on the MycoBank database (http://www.mycobank.org, accessed on 19 May 2024) and the Index Fungorum (http://www.indexfungorum.org, accessed on 19 May 2024), *Xylodon* has been registered with 234 specific and infraspecific names and the actual number of the species has reached 109 species (Chevallier 1826; Kuntze 1898; Wu 1990, 2000, 2001, 2006; Hjortstam and Ryvarden 2007, 2009; Xiong et al. 2009, 2010; Bernicchia and Gorjón 2010; Tura et al. 2011; Dai 2012; Lee and Langer 2012; Yurchenko et al. 2013; Yurchenko and Wu 2014a, b; Zhao et al. 2014; Chen et al. 2016; Kan et al. 2017a, b; Riebesehl and Langer 2017; Wang and Chen 2017; Viner et al. 2018; Riebesehl et al. 2019; Shi et al. 2019; Dai et al. 2021; Luo et al. 2021a, 2022; Qu and Zhao 2022; Qu et al. 2022; Viner and Miettinen 2022; Guan et al. 2023; Wang and Zhou 2024; Yurchenko et al. 2024; Zhang et al. 2024).

**Figure 1.**
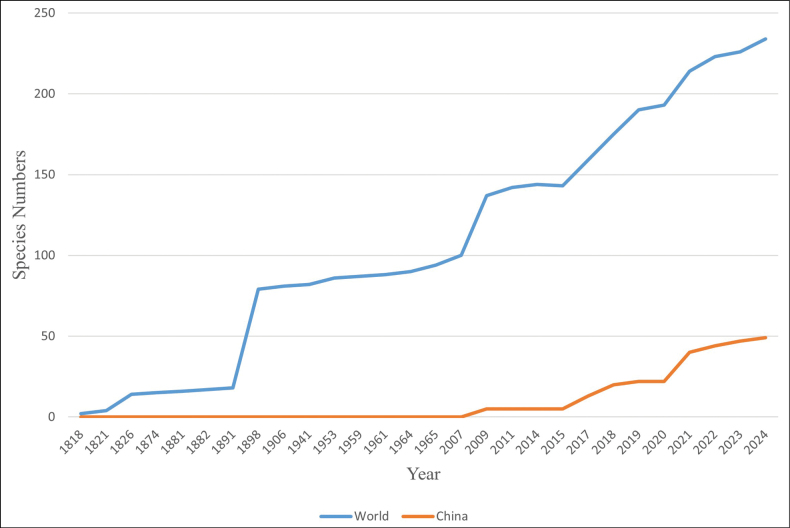
Trends in the accumulative number of species of *Xylodon* recorded in the world and China.

Classification of taxa in the kingdom *Fungi* has been updated continuously, based on the frequent inclusion of data from DNA sequences in many phylogenetic studies (Yurchenko et al. 2020). For the past few years, the genus *Xylodon* was generally studied by molecular systematics and it was included in the *Hyphodontia* s.l. (Hjortstam and Ryvarden 2009; Yurchenko and Wu 2016; Riebesehl and Langer 2017; Wang and Chen 2017; Riebesehl et al. 2019; Qu et al. 2022; Guan et al. 2023). *Hyphodontia* s.l. was shown to be a polyphyletic genus and a broad concept employed by some mycologists due to a lack of rDNA sequences for many taxa, in which *Xylodon* and *Kneiffiella* P. Karst included rich species (Hjortstam and Ryvarden 2009; Riebesehl and Langer 2017; Riebesehl et al. 2019; Luo et al. 2022; Zhang et al. 2024). Based on the molecular systematics research, two clades, the *Xylodon*-*Lyomyces*-*Rogersella* and the *Xylodon*-*Schizopora*-*Palifer* clades were described and the related species of *Lyomyces* P. Karst., *Palifer* Stalpers & P.K. Buchanan, *Rogersella* Liberta & A.J. Navas *Schizopora* Velen. and *Xylodon*, within both clades were suggested to be mixed (Yurchenko et al. 2013). The research comprised the representative sequences and taxa of *Hyphodontia* s.l., such as *Lyomyces*, *Palifer*, *Rogersella*, *Schizopora* and *Xylodon*, in which the result demonstrated that it was hard to distinguish the two genera *Xylodon* and *Schizopora* on the basis of the morphological and phylogenetic information; therefore, the authors proposed that the related species of *Schizopora* should be united into the genus *Xylodon* (Riebesehl and Langer 2017). For the phylogenetic relationship of *Xylodon* species, it was confirmed that the two genera *Lagarobasidium* Jülich and *Xylodon* should be synonymous, based on the molecular data from the ITS and nLSU regions, in which the three species *X.pumilius* (Gresl. & Rajchenb.) K.H. Larss., *X.magnificus* (Gresl. & Rajchenb.) K.H. Larss. and *X.rickii* (Gresl. & Rajchenb.) K.H. Larss. were combined into *Xylodon* (Viner et al. 2018). All the taxa of the genera *Odontipsis* Hjortstam & Ryvarden and *Palifer* were placed in the genus *Xylodon*, based on the molecular analyses of 28S and ITS data, in which they proposed four new species of *Xylodon* as *X.exilis* Yurchenko, Riebesehl & Langer, *X.filicinus* Yurchenko & Riebesehl, *X.follis* Riebesehl, Yurchenko & Langer and *X.pseudolanatus* Nakasone, Yurchenko &Riebesehl (Riebesehl et al. 2019). Based on the multiple loci in *Hyphodontia* s.l., *Fasciodontia* Yurchenko& Riebesehl, *Hastodontia* (Parmasto) Hjortstam & Ryvarden, *Hyphodontia* J. Erikss., *Lyomyces*, *Kneiffiella* and *Xylodon* in Hymenochaetales, they were divided into four clades and three new taxa were found from China, in which *X.gossypinus* C.L. Zhao & K.Y. Luo and *X.brevisetus* (P. Karst.) Hjortstam & Ryvarden grouped together (Luo et al. 2021a). Based on the morphological descriptions and molecular analyses, three new species, namely *Xylodonangustisporus* Viner & Ryvarden, *X.dissiliens* Viner & Ryvarden and *X.laxiusculus* Viner & Ryvarden, were described in Africa and placed in the genus *Xylodon* (Viner et al. 2021). A phylogenetic and taxonomic study focusing on the genus *Xylodon* (Hymenochaetales) newly described one species of this genus from southern China and this research enriched the fungal diversity worldwide (Zhang et al. 2024). Since the 1810s, a total of 234 species have been proposed for the genus *Xylodon* (http://www.indexfungorum.org/Names/Names.asp?pg=1, accessed on 19 May 2024). Inspiringly, new species have been described in the genus at an accelerated pace after the inflection point around the year 1890 and 2007 on the trend curve of species number (Fig. 1), which is due to advances in morphological taxonomy and molecular phylogeny (Luo et al. 2022; Qu et al. 2022; Guan et al. 2023; Yurchenko et al. 2024; Zhao et al. 2024; Zhang et al. 2024).

During investigations on the wood-inhabiting fungi in the Yunnan Province of China, samples representing four additional species belonging to *Xylodon* were collected. To clarify the placement and relationships of these species, we carried out a phylogenetic and taxonomic study on *Xylodon*, based on the ITS and nLSU sequences. These specimens are identified as four undescribed species of *Xylodon* and the detailed description, illustrations and phylogenetic analysis results of the new species are provided here.

## ﻿Materials and methods

### ﻿Morphology

Fresh basidiomata of the fungi growing on the angiosperm branches were collected from the Honghe, Lincang, Puer, Wenshan and Zhaotong of Yunnan Province, P.R. China. Specimens were dried in an electric food dehydrator at 40 °C (Hu et al. 2022), then sealed and stored in an envelope and deposited in the Herbarium of the Southwest Forestry University (SWFC), Kunming, Yunnan Province, P.R. China. Macromorphological descriptions were based on field notes and photos captured in the field and lab. Colour terminology followed Petersen (1996). Micromorphological data were obtained from the dried specimens when observed under a light microscope following the previous study (Guan et al. 2023). The following abbreviations are used: KOH = 5% potassium hydroxide water solution, CB = Cotton Blue, CB– = acyanophilous, IKI = Melzer’s Reagent, IKI– = both inamyloid and indextrinoid, L = mean spore length (arithmetic average for all spores), W = mean spore width (arithmetic average for all spores), Q = variation in the L/W ratios between the specimens studied and n = a/b (number of spores (a) measured from given number (b) of specimens).

### ﻿Molecular phylogeny

The EZNA HP Fungal DNA Kit (Omega Biotechnologies Co., Ltd., Kunming, China) was used to extract DNA with some modifications from the dried specimens. The nuclear ribosomal ITS region was amplified with primers ITS5 and ITS4 (White et al. 1990). The PCR procedure for ITS was as follows: initial denaturation at 95 °C for 3 min, followed by 35 cycles at 94 °C for 40 s, 58 °C for 45 s and 72 °C for 1 min, with a final extension of 72 °C for 10 min. The nuclear nLSU region was amplified with primer pair LR0R and LR7 (Rehner and Samuels 1994). The PCR procedure for nLSU was as follows: initial denaturation at 94 °C for 1 min, followed by 35 cycles at 94 °C for 30 s, 48 °C for 1 min and 72 °C for 1.5 min with a final extension of 72 °C for 10 min. The PCR procedure for ITS and nLSU followed a previous study (Zhao and Wu 2017). All newly-generated sequences were deposited in NCBI GenBank (https://www.ncbi.nlm.nih.gov/genbank/) (Table 1).

**Table 1.** Names, specimen numbers, references and corresponding GenBank accession numbers of the taxa used in this study.

Species name	Specimen No.	GenBank accession No.		References
		ITS	nLSU	
*Fasciodontiabrasiliensis*	MSK-F 7245a	MK575201	MK598734	Yurchenko et al. (2020)
*F.bugellensis*	KAS-FD 10705a	MK575203	MK598735	Yurchenko et al. (2020)
*F.bugellensis*	MSK-F 7353	MK575205	MK598736	Yurchenko et al. (2020)
*F.yunnanensis*	CLZhao 6280	MK811275	MZ146327	Luo and Zhao (2021)
*F.yunnanensis*	CLZhao 6385	MK811277	—	Luo and Zhao (2021)
*Hymenochaeteochromarginata*	He 47	KU978861	JQ279666	Unpublished
*H.rubiginosa*	He 458	JQ279580	—	He and Li (2013)
*Lyomycesalbopulverulentus*	CLZhao 21478	OP730712	OP730724	Guan et al. (2023)
*L.niveus*	CLZhao 6431	MZ262541	MZ262526	Luo et al. (2021b)
*L.niveus*	CLZhao 6442	MZ262542	MZ262527	Luo et al. (2021b)
*L.ochraceoalbus*	CLZhao 4385	MZ262535	MZ262521	Luo et al. (2021b)
*L.ochraceoalbus*	CLZhao 4725	MZ262536	MZ262522	Luo et al. (2021b)
*L.sambuci*	KAS-JR7	KY800402	KY795966	Yurchenko et al. (2017)
*L.sambuci*	83SAMHYP	JX857721	—	Yurchenko et al. (2017)
*L.yunnanensis*	CLZhao 9375	OP730710	—	Guan et al. (2023)
*L.yunnanensis*	CLZhao 10041	OP730709	—	Guan et al. (2023)
*X.asiaticus*	CLZhao 10368	OM959479	—	Zhang et al. (2024)
*X.attenuatus*	Spirin 8775	MH324476	—	Wang et al. (2021)
*X.asperus*	Spirin 11923	OK273838	—	Viner et al. (2021)
*X.apacheriensis*	Canfield 180	KY081800	—	Wang et al. (2021)
*X.acuminatus*	Larsson 16029	ON197552	—	Viner et al. (2023)
*X.acystidiatus*	LWZ 20180514-9	MT319474	—	Wang et al. (2021)
*X.afromontanus*	H 7006811	OQ645463	—	Yurchenko et al. (2024)
*X.angustisporus*	Ryvarden 50691b	OK273831	—	Viner et al. (2021)
*X.astrocystidiatus*	TNM F24764	NR154054	—	Yurchenko and Wu (2014b)
*X.australis*	LWZ 20180509-8	MT319503	—	Wang et al. (2021)
*X.bambusicola*	CLZhao 11310	MW394660	—	Ma and Zhao (2021)
*X.borealis*	JS 26064	AY463429	—	Larsson et al. (2004)
*X.brevisetus*	JS 17863	AY463428	—	Larsson et al. (2004)
***X.cremeoparinaceus***	**CLZhao 23388**	**PP537951**	—	**Present study**
*X.crystalliger*	KUN 2312	NR166242	—	Viner et al. (2018)
*X.cymosus*	Miettinen 19606	ON197554	—	Viner et al. (2023)
*X.cystidiatus*	FR-0249200	MH880195	—	Wang et al. (2021)
*X.damansaraensis*	LWZ 20180417-23	MT319499	—	Wang et al. (2021)
*X.daweishanensis*	CLZhao 18357	OP730715	—	Guan et al. (2023)
*X.detriticus*	Zíbarová 30.10.17	MH320793	—	Wang et al. (2021)
*X.dissiliens*	Ryvarden 44817	OK273856	—	Viner et al. (2021)
*X.echinatus*	OM 18237	OQ645464	—	Yurchenko et al. (2024)
*X.filicinus*	MSK-F 12869	MH880199	—	Wang et al. (2021)
*X.fissuratus*	CLZhao 9407	OP730714	—	Guan et al. (2023)
*X.flaviporus*	FR-0249797	MH880201	—	Wang et al. (2021)
*X.flocculosus*	CLZhao 18342	MW980776	—	Qu and Zhao (2022)
*X.follis*	FR-0249814	MH880204	—	Wang et al. (2021)
*X.gloeocystidiifer*	BLS M-5232	OQ645467	—	Yurchenko et al. (2024)
*X.gossypinus*	CLZhao 8375	MZ663804	—	Luo et al. (2021a)
*X.grandineus*	CLZhao 6425	OM338090	—	Luo et al. (2022)
*X.hastifer*	K(M) 172400	NR166558	—	Riebesehl and Langer (2017)
*X.heterocystidiatus*	Wei 17-314	MT731753	—	Unpublished
*X.hjortstamii*	Gorjon 3187	ON188816	—	Unpublished
*X.hyphodontinus*	KAS-GEL9222	MH880205	—	Riebesehl et al. (2019)
*X.jacobaeus*	MA-Fungi 91340	MH430073	—	Wang et al. (2021)
*X.kunmingensis*	TUB-FO 42565	MH880198	—	Wang et al. (2021)
*X.laceratus*	CLZhao 9892	OL619258	—	Qu et al. (2022)
*X.lagenicystidiatus*	LWZ 20180515-14	MT319633	—	Wang et al. (2021)
*X.lagenicystidiatus*	LWZ 20180513-16	MT319634	—	Wang et al. (2021)
*X.lanatus*	CFMR FP-101864-A	OQ645474	—	Yurchenko et al. (2024)
*X.laxiusculus*	Ryvarden 44877	OK273827	—	Viner et al. (2021)
*X.lenis*	Wu 890714-3	KY081802	—	Yurchenko et al. (2024)
***X.luteodontioides***	**CLZhao 3207**	**MH114740**	—	**Present study**
***X.luteodontioides***	**CLZhao 18494**	**PP505422**	—	**Present study**
*X.macrosporus*	CLZhao 10226	MZ663809	—	Luo et al. (2021a)
*X.magallanesii*	MA: Fungi:90397	MT158729	—	Fernandez-Lopez et al. (2020)
*X.mantiqueirensis*	MV 529	OQ645478	—	Yurchenko et al. (2024)
*X.mollissimus*	LWZ 20160318-3	KY007517	—	Kan et al. (2017)
*X.montanus*	CLZhao 8179	OL619260	—	Qu et al. (2022)
*X.neotropicus*	MV 580	OQ645479	—	Yurchenko et al. (2024)
*X.nesporii*	LWZ 20180921-35	MT319655	—	Wang et al. (2021)
*X.nesporii*	LWZ 20190814-17a	ON063679	—	Wang et al. (2023)
*X.niemelaei*	LWZ 20150707-13	MT319630	—	Wang et al. (2021)
*X.nongravis*	GC 1412-22	KX857801	—	Wang et al. (2021)
*X.nothofagi*	ICMP 13842	AF145583	—	Wang et al. (2021)
*X.ovisporus*	LWZ 20170815-31	MT319666	—	Wang et al. (2021)
*X.papillosus*	CBS 114.71	MH860026	—	Vu et al. (2019)
*X.paradoxus*	Dai 14983	MT319519	—	Wang et al. (2021)
*X.patagonicus*	ICMP 13832	AF145581	—	Wang et al. (2021)
***X.poroides***	**CLZhao 17845**	**PP505420**	**PP657608**	**Present study**
*X.pruinosus*	Spirin 2877	MH332700	—	Wang et al. (2021)
*X.pruniaceus*	Ryvarden 11251	OK273828	—	Viner et al. (2021)
*X.pseudolanatus*	FP-150922	MH880220	—	Wang et al. (2021)
*X.pseudotropicus*	Dai 10768	KF917543	—	Wang et al. (2021)
*X.pseudotropicus*	Dai 16167	MT326536	—	Wang et al. (2021)
*X.puerensis*	CLZhao 8142	OP730720	—	Guan et al. (2023)
*X.punctus*	CLZhao 17691	OM338092	—	Luo et al. (2022)
*X.punctus*	CLZhao 17908	OM338093	—	Luo et al. (2022)
*X.punctus*	CLZhao 17916	OM338094	—	Luo et al. (2022)
*X.quercinus*	Spirin 12030	OK273841	—	Viner et al. (2021)
*X.raduloides*	FCUG 2433	AF145570	—	Wang et al. (2021)
*X.ramicida*	Spirin 7664	NR138013	—	Unpublished
*X.reticulatus*	Wu 1109-178	KX857805	—	Wang et al. (2021)
*X.reticulatus*	GC 1512-1	KX857808	—	Wang et al. (2021)
*X.rimosissimus*	Ryberg 021031	DQ873627	—	Wang et al. (2021)
*X.rhizomorphus*	Dai 12367	NR154067	—	Zhao et al. (2014)
*X.rhododendricola*	LWZ 20180513-9	MT319621	—	Wang et al. (2021)
*X.serpentiformis*	LWZ 20170816-15	MT319673	—	Wang et al. (2021)
*X.sinensis*	CLZhao 9197	MZ663810	—	Luo et al. (2021a)
*X.sinensis*	CLZhao 11120	MZ663811	—	Luo et al. (2021a)
*X.spathulatus*	LWZ 20180804-10	MT319646	—	Wang et al. (2021)
*X.subclavatus*	FO 42167	MH880232	—	Wang et al. (2021)
*X.subflaviporus*	TNM F29958	NR184880	—	Chen et al. (2017)
*X.submucronatus*	Renvall 1602	OK273830	—	Viner et al. (2021)
*X.subserpentiformis*	LWZ 20180512-16	MT319486	—	Wang et al. (2021)
*X.subtilissimus*	Spirin 12228	ON188818	—	Unpublished
*X.subtropicus*	LWZ 20180510-24	MT319541	—	Wang et al. (2021)
*X.taiwanianus*	CBS 125875	MH864080	—	Vu et al. (2019)
*X.tropicus*	CLZhao 3351	OL619261	—	Qu et al. (2022)
*X.ussuriensis*	KUN 1989	NR166241	—	Unpublished
*X.verecundus*	KHL 12261	DQ873642	—	Wang et al. (2021)
*X.victoriensis*	LWZ 20180510-29	MT319487	—	Wang et al. (2021)
*X.wenshanensis*	CLZhao 15729	OM338097	—	Luo et al. (2022)
***X.wumengshanensis***	**CLZhao 32517**	**PP645439**	**PP826351**	**Present study**
*X.xinpingensis*	CLZhao 9174	MW394657	—	Ma and Zhao (2021)
*X.yarraensis*	LWZ 20180510-5	MT319639	—	Wang et al. (2021)
*X.yunnanensis*	LWZ 20180922-47	MT319660	—	Wang et al. (2021)

**Figure 2.**
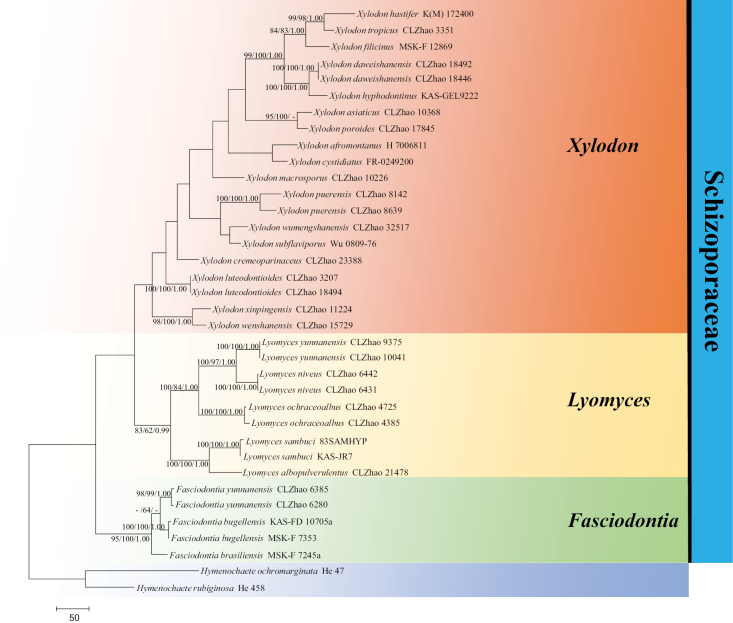
Maximum Parsimony strict consensus tree illustrating the phylogeny of four new species and related species in *Xylodon* within Schizoporaceae, based on ITS+nLSU sequences. Branches are labelled with Maximum Likelihood bootstrap values ≥ 70%, parsimony bootstrap values ≥ 50% and Bayesian posterior probabilities ≥ 0.95, respectively.

**Figure 3.**
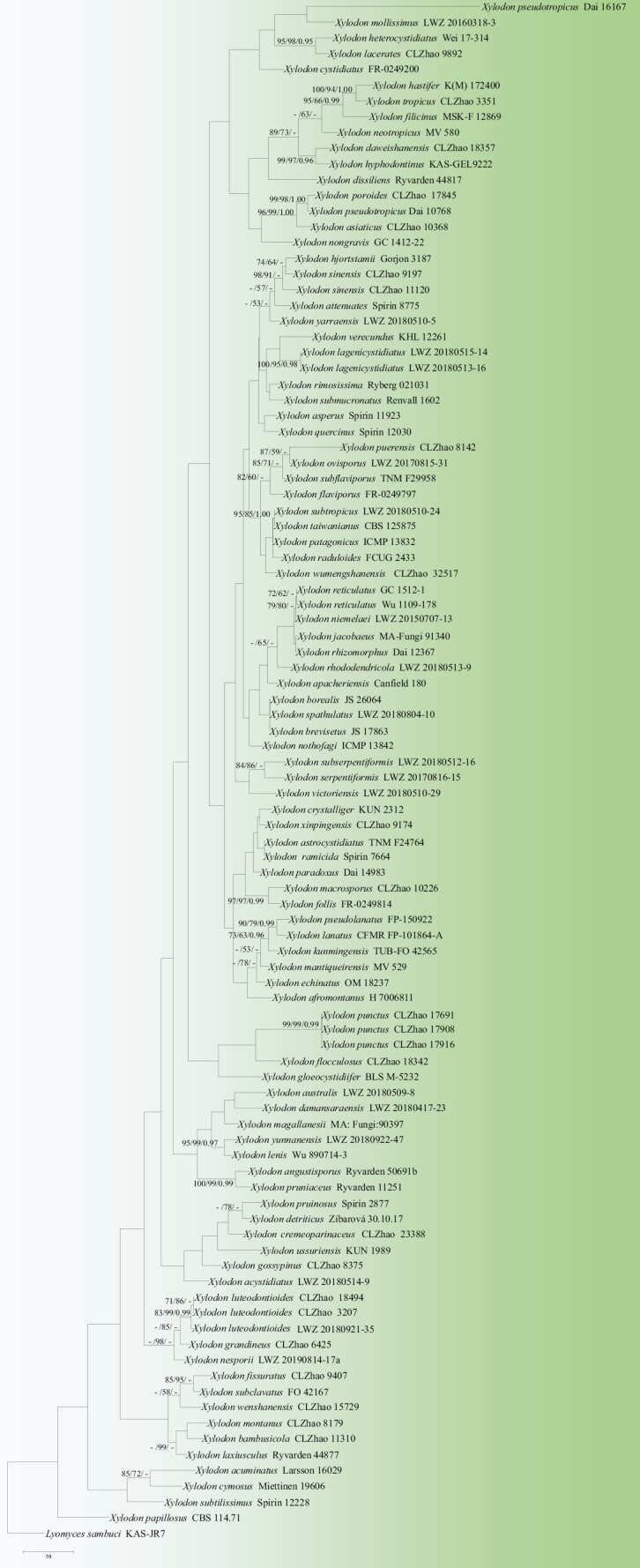
Maximum parsimony strict consensus tree illustrating the phylogeny of the four new species and related species in *Xylodon*, based on ITS sequences. Branches are labelled with Maximum Likelihood bootstrap values ≥ 70%, parsimony bootstrap values ≥ 50% and Bayesian posterior probabilities ≥ 0.95, respectively.

The sequences were aligned in MAFFT version 7 (Katoh et al. 2019) using the G-INS-i strategy. The alignment was adjusted manually using AliView version 1.27 (Larsson 2014). Sequences of *Hymenochaeteochromarginata* P.H.B. Talbot and *H.rubiginosa* (Dicks.) Lév. retrieved from GenBank were used as the outgroups in the ITS+nLSU analysis (Fig. 2); Sequences of *Lyomycessambuci* (Pers.) P. Karst. retrieved from GenBank were used as the outgroups in the ITS analysis (Fig. 3) (Guan et al. 2023; Zhang et al. 2024).

Maximum Parsimony (MP), Maximum Likelihood (ML) and Bayesian Inference (BI) analyses were applied to the combined three datasets following a previous study (Zhao and Wu 2017). All characters were equally weighted and gaps were treated as missing data. Trees were inferred using the heuristic search option with TBR branch swapping and 1,000 random sequence additions. Max trees were set to 5000, branches of zero length were collapsed and all parsimonious trees were saved. Clade robustness was assessed using bootstrap (BT) analysis with 1000 pseudo replicates (Felsenstein 1985). Descriptive tree statistics - tree length (TL), composite consistency index (CI), composite retention index (RI), composite rescaled consistency index (RC) and composite homoplasy index (HI) - were calculated for each maximum parsimonious tree generated. The combined dataset was also analysed using Maximum Likelihood (ML) in RAxML-HPC2 through the CIPRES Science Gateway (Miller et al. 2012). Branch support (BS) for the ML analysis was determined by 1000 bootstrap pseudo replicates.

MrModelTest 2.3 (Nylander 2004) was used to determine the best-ﬁt evolution model for each dataset for the purposes of Bayesian Inference (BI) which was performed using MrBayes 3.2.7a with a GTR+I+G model of DNA substitution and a gamma distribution rate variation across sites (Ronquist et al. 2012). A total of four Markov chains were run for two runs from random starting trees for 1.3 million generations for ITS+nLSU (Fig. 2) and 16 million generations for ITS (Fig. 3) with trees and parameters sampled every 1,000 generations. The ﬁrst quarter of all of the generations were discarded as burn-in. A majority rule consensus tree was computed from the remaining trees. Branches were considered as significantly supported if they received a Maximum Likelihood bootstrap support value (BS) of ≥ 70%, a maximum parsimony bootstrap support value (BT) of ≥ 70% or a Bayesian posterior probability (BPP) of ≥ 0.95.

## ﻿Results

### ﻿Molecular phylogeny

The ITS+nLSU dataset (Fig. 2) comprised sequences from 36 fungal specimens representing 27 taxa. The dataset had an aligned length of 2130 characters, of which 1312 characters were constant, 301 were variable and parsimony-uninformative and 517 were parsimony-informative. Maximum parsimony analysis yielded three equally parsimonious trees (TL = 2445, CI = 0.5051, HI = 0.4949, RI = 0.6113 and RC = 0.3088). The best model of nucleotide evolution for the ITS+nLSU dataset estimated and applied in the Bayesian analysis was found to be GTR+I+G. Bayesian analysis and ML analysis resulted in a similar topology as in the MP analysis. The Bayesian analysis had an average standard deviation of split frequencies = 0.005704 (BI) and the effective sample size (ESS) across the two runs is double the average ESS (avg. ESS) = 337. The phylogram, based on the ITS+nLSU rDNA gene regions (Fig. 2), included three genera viz. *Fasciodontia*, *Lyomyces* and *Xylodon*, within the family Schizoporaceae (Hymenochaetales), in which four new species were grouped into the genus *Xylodon*.

The ITS+nLSU dataset (Fig. 3) comprised sequences from 98 fungal specimens representing 88 taxa. The dataset had an aligned length of 748 characters, of which 219 characters were constant, 168 were variable and parsimony-uninformative and 361 were parsimony-informative. Maximum parsimony analysis yielded 100 equally parsimonious trees (TL = 3719, CI = 0.2533, HI = 0.7467, RI = 0.4268 and RC = 0.1081). The best model of nucleotide evolution for the ITS dataset estimated and applied in the Bayesian analysis was found to be GTR+I+G. Bayesian analysis and ML analysis resulted in a similar topology as in the MP analysis. The Bayesian analysis had an average standard deviation of split frequencies = 0.015679 (BI) and the effective sample size (ESS) across the two runs is double the average ESS (avg. ESS) = 3443. The phylogenetic tree (Fig. 3), inferred from the ITS+nLSU sequences, highlighted that *X.cremeoparinaceus* was grouped closely with *X.pruinosus* (Bres.) Spirin & Viner, *X.detriticus* (Bourdot) K.H. Larss., Viner & Spirin and *X.ussuriensis* Viner. The taxon *X.luteodontioides* was sister to *X.nesporii* (Bres.) Hjortstam & Ryvarden. The species *X.poroides* was sister to *X.pseudotropicus* (C.L. Zhao, B.K. Cui & Y.C. Dai) Riebesehl, Yurch. & Langer. The species *X.wumengshanensis* was grouped with four taxa: *X.patagonicus* J. Fernández-López, Telleria, M. Dueñas & M.P. Martín, *X.radula* (Fr.) Ţura, Zmitr., Wasser & Spirin, *X.subtropicus* (Che C. Chen & Sheng H. Wu) Che C. Chen & Sheng H. Wu and *X.taiwanianus* (Sheng H. Wu) Hjortstam & Ryvarden.

### ﻿Taxonomy

#### ﻿ Xylodon cremeoparinaceus

Taxon classificationFungiHymenochaetalesSchizoporaceae

Q. Yuan & C.L. Zhao sp. nov.

B9746559-FC63-5C09-97F0-1C9955977630

 854061

Figs 4 , 5

##### Holotype.

China. Yunnan Province, Zhaotong, Zhaoyang District, Fenghuang Mountain Forest Park, GPS coordinates 27°29'N, 103°68'E, altitude 2872 m, on the fallen branch of angiosperm, leg. C.L. Zhao, 24 August 2022, CLZhao 23388 (SWFC).

**Figure 4.**
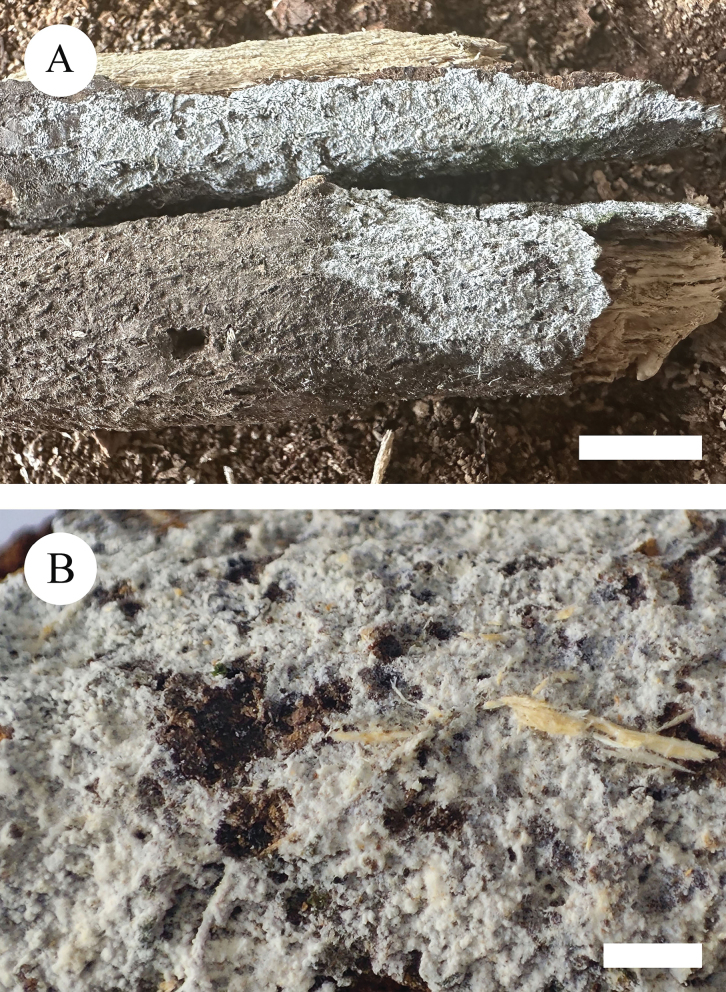
Basidiomata of *Xylodoncremeoparinaceus* (holotype). Scale bars: 1 cm (**A**); 2 mm (**B**).

##### Etymology.

*cremeoparinaceus* (Lat.): referring to the cream hymenial surface with pruinose hymenophore.

**Figure 5.**
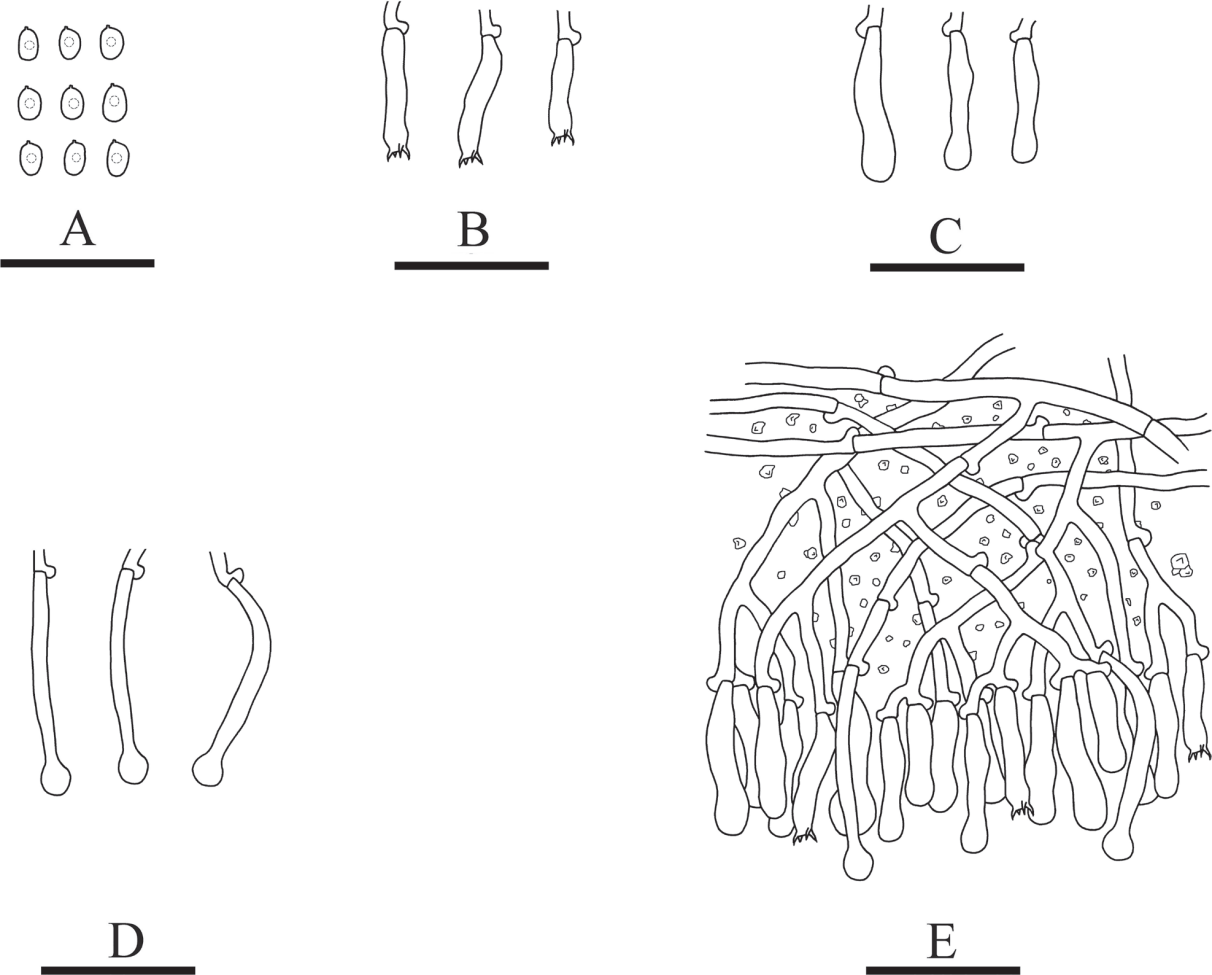
Microscopic structures of *Xylodoncremeoparinaceus* (holotype): basidiospores (**A**), basidia (**B**), basidioles (**C**), capitate cystidia (**D**), a section of hymenium (**E**). Scale bars: 20 µm (**A–E**).

##### Description.

Basidiomata annual, resupinate, adnate, farinaceous, without odour or taste when fresh, up to 2.5 cm long, 1.5 cm wide, 50–80 um thick. Hymenial surface reticulate, white to cream when fresh, turning to cream upon drying. Sterile margin white, up to 1 mm wide.

Hyphal system monomitic, generative hyphae with clamp connections, colourless, thin-walled, frequently branched, interwoven, 1.5–2 µm in diameter; IKI–, CB–, tissues unchanged in KOH. Numerous crystals present amongst generative hyphae.

Cystidia capitate, colourless, thin-walled, smooth, slightly constricted at the neck, with a globose tip, 18.5–25 × 3.5–6.5 µm; basidia subclavate, with 4 sterigmata and a basal clamp connection, 13.5–17.5 × 3–3.5 µm.

Basidiospores ellipsoid, colourless, thin-walled, smooth, with one drop, cyanophilous, IKI–, 3.5–4.5 × 2.5–3.5 µm, L = 3.71 µm, W = 2.82 µm, Q = 1.31 (n = 30/1).

#### ﻿ Xylodon luteodontioides

Taxon classificationFungiHymenochaetalesSchizoporaceae

Q. Yuan & C.L. Zhao sp. nov.

47CB6558-699A-5F11-8897-8BAF06D8DA66

 854060

Figs 6 , 7

##### Holotype.

China. Yunnan Province, Puer, Laiyanghe National Forestry Park, GPS coordinates 22°60'N, 101°00'E, altitude 1500 m, on the fallen branch of angiosperm, leg. C.L. Zhao, 30 September 2017, CLZhao 3207 (SWFC).

**Figure 6.**
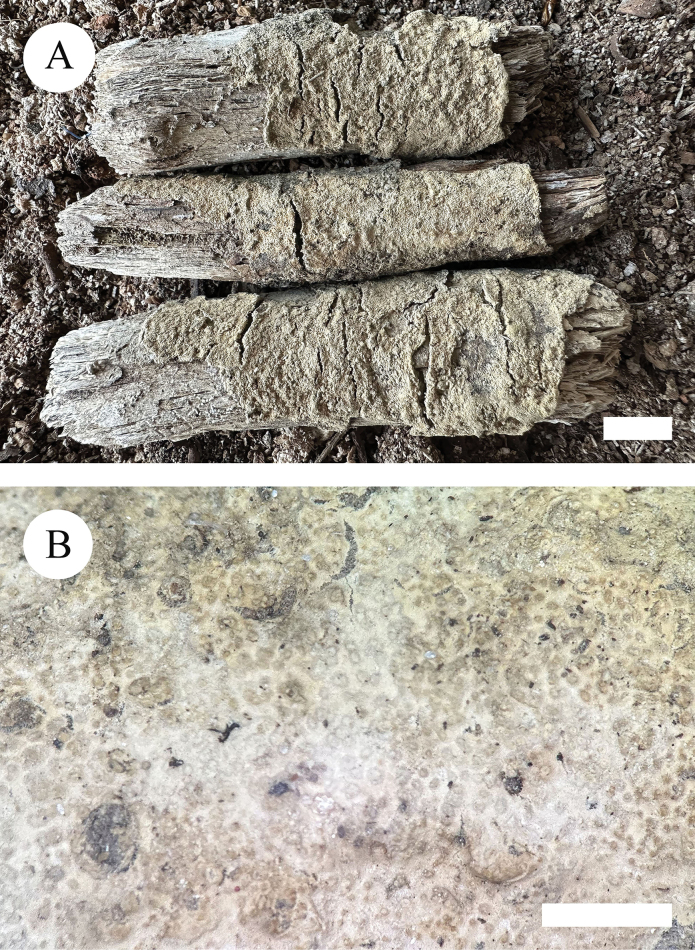
Basidiomata of *Xylodonluteodontioides* (holotype). Scale bars: 1 cm (**A**); 2 mm (**B**).

##### Etymology.

*luteodontioides* (Lat.): referring to the flavescent hymenophore surface with odontioid hymenophore.

**Figure 7.**
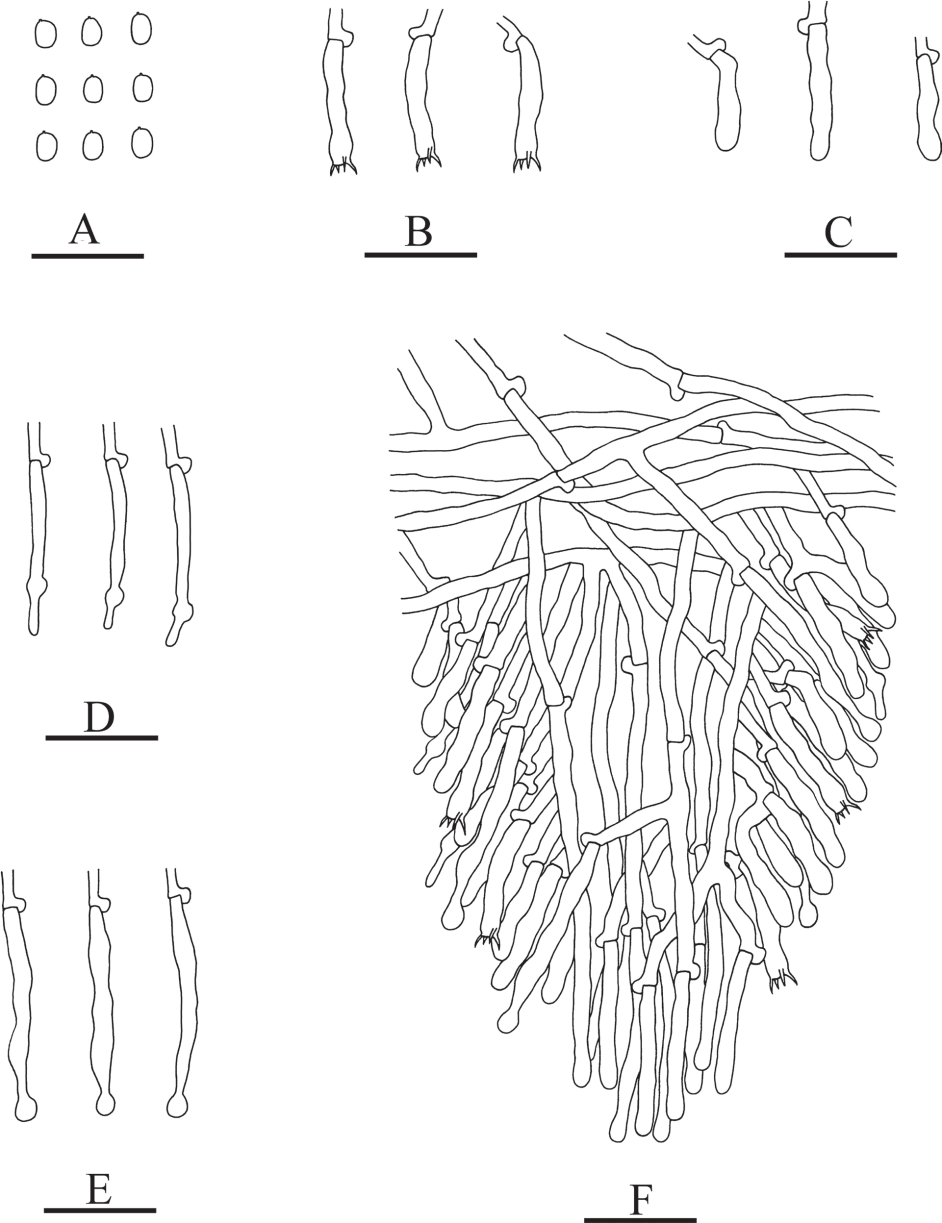
Microscopic structures of *Xylodonluteodontioides* (holotype): basidiospores (**A**), basidia (**B**), basidioles (**C**), schizopapillate cystidia (**D**), capitate cystidia (**E**), a section of hymenium (**F**). Scale bars: 20 µm (**A–F**).

##### Description.

Basidiomata annual, resupinate, adnate, coriaceous, without odour and taste when fresh and up to 7 cm long, 4 cm wide, 100 μm thick. Hymenial surface odontioid, buff when fresh, to buff to olivaceous-buff upon drying. Sterile margin slightly buff and up to 1 mm wide.

Hyphal system monomitic, generative hyphae with clamp connections, colourless, thin-walled, branched, 2.5–3.5 µm in diameter; IKI–, CB–, tissues unchanged in KOH.

Cystidia of two types: (1) schizopapillate cystidia, colourless, thin-walled, smooth, 29.5–37 × 2.5–3.5 µm; (2) capitate cystidia, colourless, thin-walled, smooth, 38.5–44.5 × 3.5–4 µm; basidia subclavate, with 4 sterigmata and a basal clamp connection, 19.5–26 × 3.5–4 µm.

Basidiospores ellipsoid, colourless, thin-walled, smooth, CB–, IKI–, 3.5–4.5 × 2.5–3.5 µm, L = 4.07 µm, W = 2.92 µm, Q = 1.39–1.45 (n = 60/2).

##### Additional specimens examined

**(*paratypes*).** China. Yunnan Province, Honghe, Pingbian Country, Daweishan National Nature Reserve, GPS coordinates 22°93'N, 103°69'E, altitude 1800 m, on the fallen branch of angiosperm, leg. C.L. Zhao, 3 August 2019, CLZhao 18494 (SWFC).

#### ﻿ Xylodon poroides

Taxon classificationFungiHymenochaetalesSchizoporaceae

Q. Yuan & C.L. Zhao sp. nov.

1B4FD918-5E91-54A6-88DE-89FA1182EE7F

 854059

Figs 8 , 9

##### Holotype.

China. Yunnan Province, Honghe, Pingbian Country, Daweishan National Nature Reserve, GPS coordinates 22°93'N, 103°69'E, altitude 1800 m, on the fallen branch of angiosperm, leg. C.L. Zhao, 1 August 2019, CLZhao 17845 (SWFC).

**Figure 8.**
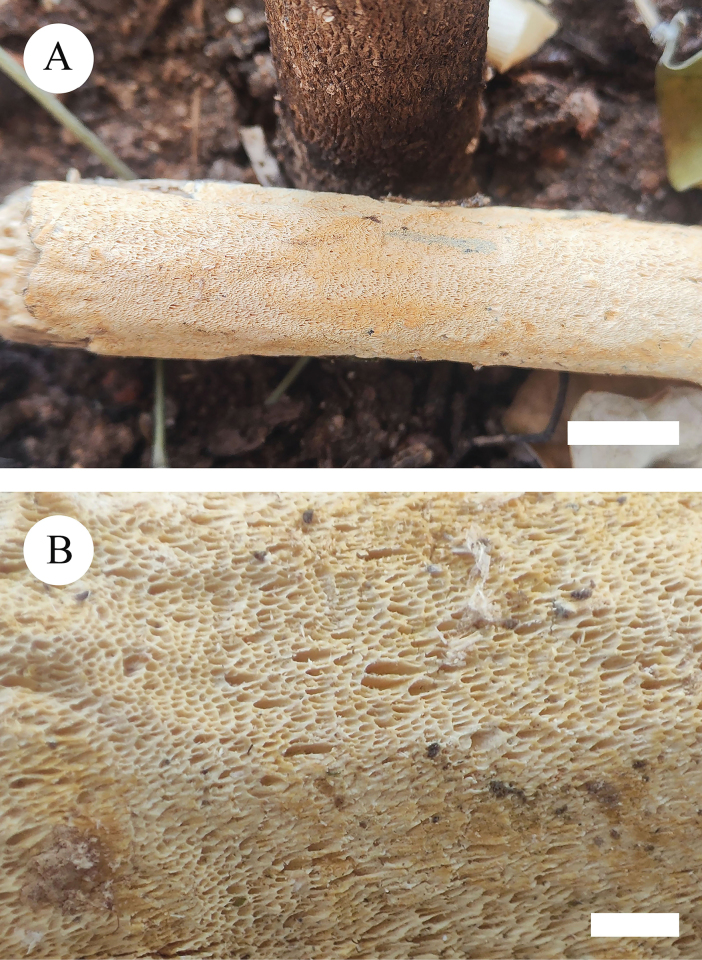
Basidiomata of *Xylodonporoides* (holotype). Scale bars: 1 cm (**A**); 2 mm (**B**).

##### Etymology.

*poroides* (Lat.): referring to the poroid hymenophore surface.

**Figure 9.**
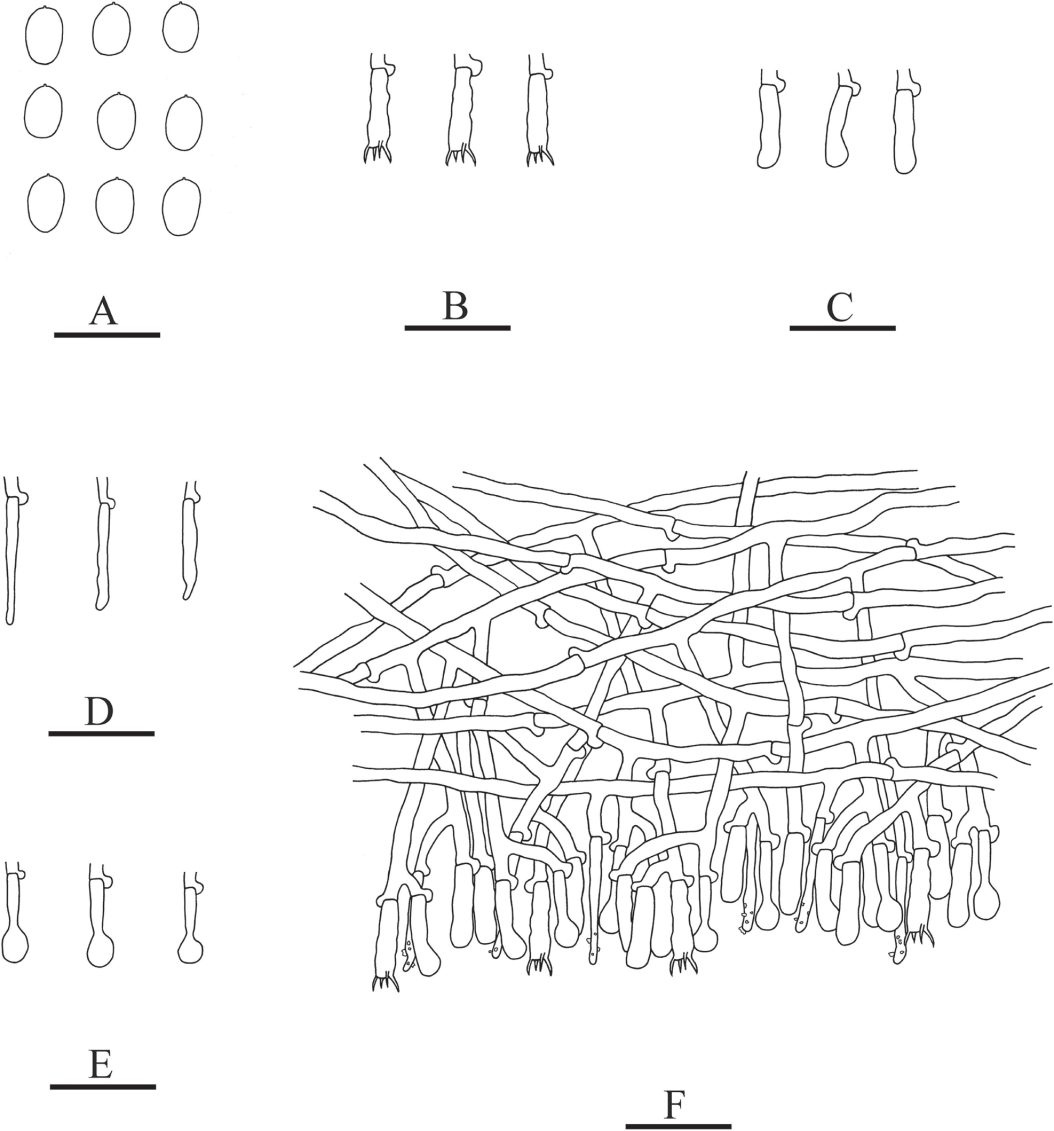
Microscopic structures of *Xylodonporoides* (holotype): basidiospores (**A**), basidia (**B**), basidioles (**C**), fusoid cystidia (**D**), capitate cystidia (**E**), a section of hymenium (**F**). Scale bars: 10 µm (**A**); 20 µm (**B–F**).

##### Description.

Basidiomata annual, resupinate, adnate, coriaceous, without odour and taste when fresh and up to 14 cm long, 4 cm wide, 200 µm thick. Hymenial surface poroid, pores angular, 4–5 per mm, cream to pink-buff when fresh, turn to flesh-pink to pink-buff upon drying. Sterile margin slightly buff and up to 1 mm wide.

Hyphal system monomitic, generative hyphae with clamp connections, colourless, thin-walled, frequently branched, 2–3 µm in diameter; IKI–, CB–, tissues unchanged in KOH.

Cystidia of two types: (1) fusoid cystidia, colourless, thin-walled, smooth, 17.5–24.5 × 2.5–3 µm, encrusted crystals; (2) capitate cystidia, colourless, thin-walled, smooth, 11.5–15.5 × 3.5–4.5 µm; basidia clavate, with 4 sterigmata and a basal clamp connection, 15.5–19 × 3.5–5.5 µm.

Basidiospores ellipsoid, colourless, thin-walled, smooth, CB–, IKI–, (3.5–)4–5.5 × 2.5–3.5(–5) µm, L = 4.82 µm, W = 2.95 µm, Q = 1.63 (n = 30/1).

#### ﻿ Xylodon wumengshanensis

Taxon classificationFungiHymenochaetalesSchizoporaceae

Q. Yuan & C.L. Zhao sp. nov.

D8223AC3-1CE3-546B-B3D3-91638809A0A2

 854058

Figs 10 , 11

##### Holotype.

China. Yunnan Province, Zhaotong, Wumengshan National Nature Reserve, GPS coordinates 27°77'N, 104°29'E, altitude 2900 m, on the fallen branch of angiosperm, leg. C.L. Zhao, 29 August 2023, CLZhao 32517 (SWFC).

**Figure 10.**
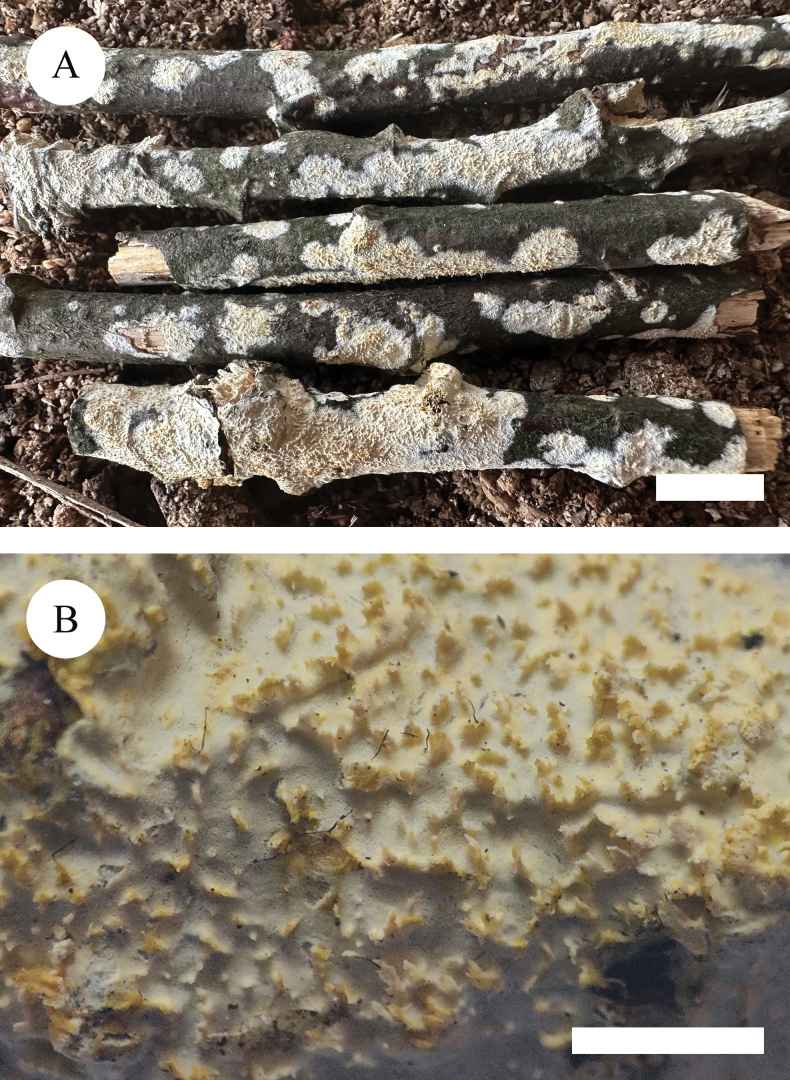
Basidiomata of *Xylodonwumengshanensis* (holotype). Scale bars: 1 cm (**A**); 2 mm (**B**).

##### Etymology.

*wumengshanensis* (Lat.): referring to the locality (Wumengshan) of the type specimen.

**Figure 11.**
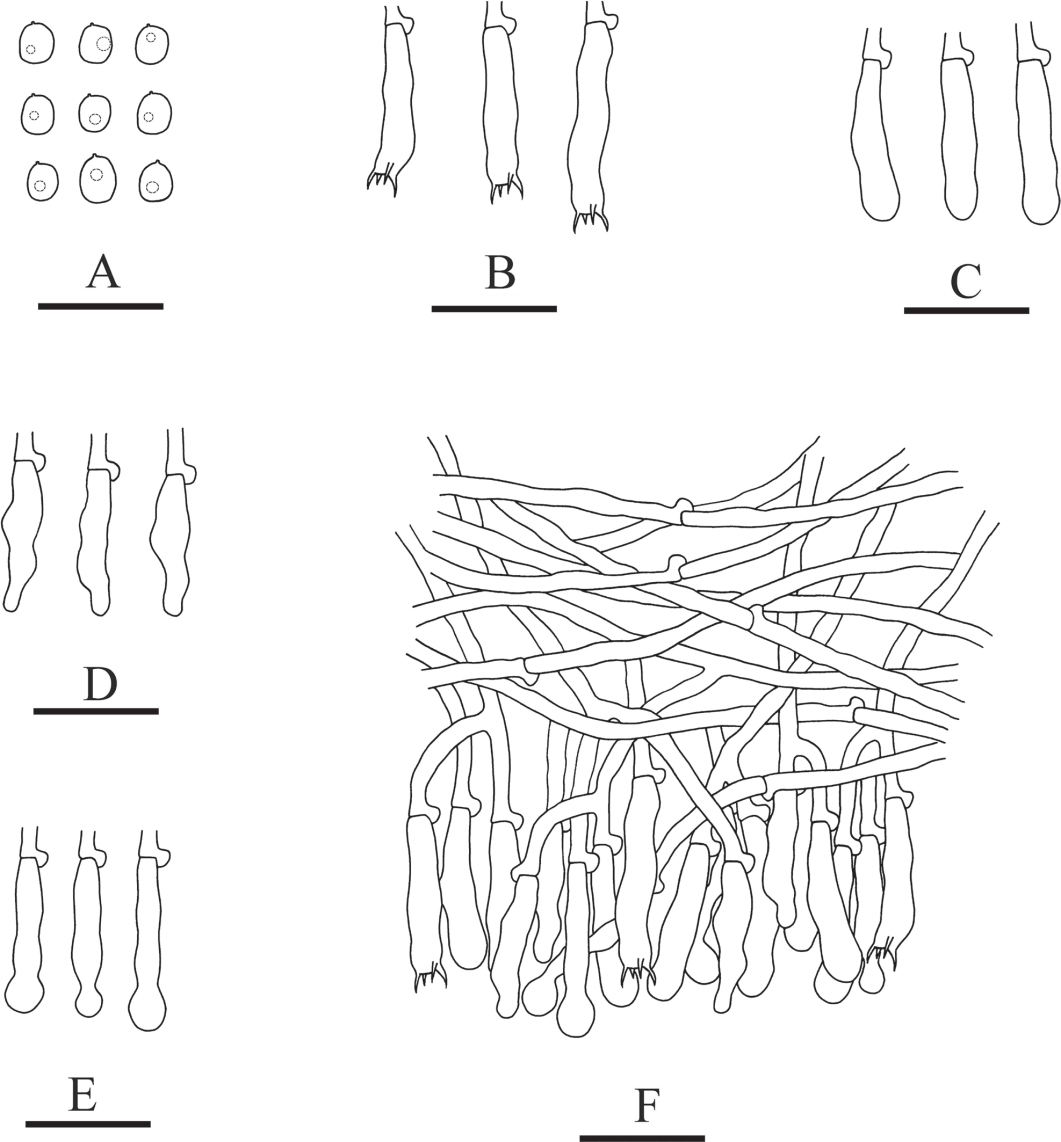
Microscopic structures of *Xylodonwumengshanensis* (holotype): basidiospores (**A**), basidia (**B**), basidioles (**C**), fusoid cystidia (**D**), capitate cystidia (**E**), a section of hymenium (**F**). Scale bars: 20 µm (**A**–**F**).

##### Description.

Basidiomata annual, resupinate, adnate, coriaceous, without odour and taste when fresh and up to 4.5 cm long, 1.5 cm wide, 100–200 µm thick. Hymenial surface grandinoid, cream when fresh; turn to cream to buff upon drying. Sterile margin distinct, cream, up to 1 mm wide.

Hyphal system monomitic, generative hyphae with clamp connections, colourless, thick-walled, rarely branched, 2–3.5 µm in diameter; IKI–, CB–, tissues unchanged in KOH.

Cystidia of two types: (1) capitate, colourless, thin-walled, smooth, slightly constricted at the neck, with a globose head, 24.5–29.5 × 5–6 µm; (2) fusoid, colourless, thin-walled, smooth, 14.5–22 × 5.5–6.5 µm; basidia clavate, with 4 sterigmata and a basal clamp connection, 22.5–33 × 5–5.5 µm.

Basidiospores ellipsoid, colourless, thin-walled, smooth, with one oil drop, CB–, IKI–, (4.5–) 5–6.5(–7) × 4–5.5 µm, L = 5.55 µm, W = 4.39 µm, Q = 1.26 (n = 30/1).

## ﻿Discussion

Many recently-described wood-inhabiting fungal taxa have been reported in the subtropics and tropics, including in the genus *Xylodon* (Xiong et al. 2009; Chen et al. 2017; Kan et al. 2017a, b; Riebesehl and Langer 2017; Viner et al. 2018; Chen and Zhao 2020; Luo et al. 2021a, b, c; Luo et al. 2022; Qu and Zhao 2022; Qu et al. 2022; Viner and Miettinen 2022; Guan et al. 2023; Deng et al. 2024a, b; Zhang et al. 2024). Prior to this study, the following forty-five *Xylodon* species were reported from China. The present study reports four new species in *Xylodon*, based on a combination of morphological features and molecular evidence.

Phylogenetically, based on the multiple loci in Schizoporaceae, three genera *Fasciodontia*, *Lyomyces* and *Xylodon* were located in this family (Wang et al. 2021). In the present study, the phylogram inferred from the ITS+nLSU data, four new species grouped into the genus *Xylodon* (Fig. 2). Based on ITS topology (Fig. 3), *Xylodoncremeoparinaceus* grouped closely with three species viz. *X.detriticus*, *X.pruinosus* and *X.ussuriensis*. The taxon *X.luteodontioides* was sister to *X.nesporii*. The species *X.poroides* was sister to *X.pseudotropicus*. The taxon *X.wumengshanensis* grouped with four taxa viz. *X.patagonicus*, *X.radula*, *X.subtropicus* and *X.taiwanianus*. However, morphologically, *X.detriticus* can be delimited from *X.cremeoparinaceus* by its smooth or warted, farinaceous hymenial surface and its wider basidia (13.1–20.0 × 3.4–5.0 µm; Viner et al. (2018)); *X.pruinosus* can be delimited from *X.cremeoparinaceus* by its grandinioid to odontoid hymenial surface and its larger basidiospores (4.5–5.9 × 3.7–4.8 µm; Viner et al. (2018)); *X.ussuriensis* can be delimited from *X.cremeoparinaceus* by its grandinioid to odontoid hymenial surface and its larger basidiospores (5.1–6.0 × 3.8–4.6 µm; Viner et al. (2018)). *Xylodonnesporii* can be delimited from *X.luteodontioides* by its subcylindrical basidia (15–25 × 4–5 µm) and its longer and narrower basidiospores (4.5–6 × 2–2.5 µm; Hjortstam and Ryvarden (2009)); *X.pseudotropicus* can be delimited from *X.poroides* by its shorter basidia (9–12.5 × 3–5 µm) and its oblong-ellipsoid basidiospores (4.3–4.9 × 2.8–3 µm; Zhao et al. (2014)); *X.patagonicus* can be delimited from *X.wumengshanensis* by its poroid to labyrinthiform hymenial surface and its narrower basidiospores (4–5.5 × 2.5–3.5 µm; Fernández-López et al. (2019)); *X.radula* can be delimited from *X.wumengshanensis* by its subclavate to subcylindrical basidia (20–25 × 4–6 µm) and its longer and narrower basidiospores (9–11 × 3–3.5 µm; Eriksson and Ryvarden (1975)); *X.subtropicus* can be delimited from *X.wumengshanensis* by its poroid hymenial surface and its smaller basidiospores (13–18 × 4–4.5 μm; Chen et al. (2018)); *X.taiwanianus* can be delimited from *X.wumengshanensis* by its poroid hymenial surface and its smaller basidia 14–20 × 4–5 μm; Wu (2001)).

Morphologically, *Xylodoncremeoparinaceus* resembles *X.fissuratus*, *X.flocculosus*, *X.grandineus*, *X.laceratus*, and *X.wenshanensis* K.Y. Luo & C.L. Zhao by ellipsoid basidiospores. However, *X.fissuratus* differs from *X.cremeoparinaceus* due to its grandinioid hymenial surface and its shorter capitate cystidia (11.5–16.5 × 3–4.5 µm; Guan et al. (2023)); *X.flocculosus* differs from *X.cremeoparinaceus* due to its grandinoid hymenial surface and its barrel-shaped basidia (11–20 × 3.3–4.8 µm; Qu and Zhao (2022)); *X.grandineus* differs from *X.cremeoparinaceus* due to its grandinioid hymenial surface and by possessing subulate cystidia (11–19 × 3–5 µm; Luo et al. (2022)); *X.laceratus* differs from *X.cremeoparinaceus* due to its grandinioid hymenial surface and by possessing fusiform cystidia (20.3–26.8 × 5.3–6.4 µm; Qu et al. (2022)); *X.wenshanensis* differs from *X.cremeoparinaceus* due to its grandinioid hymenial surface and its shorter cystidia (6–11 × 3–6.5 µm; Luo et al. (2022)).

Morphologically, *X.luteodontioides* resembles *X.fissuratus*, *X.laurentianus* J. Fernández-López, Telleria, M. Dueñas & M.P. Martín, *X.puerensis* C.L. Zhao, *X.subflaviporus* Che C. Chen & Sheng H. Wu and *X.wenshanensis* due to the capitate cystidia. However, *X.fissuratus* differs from *X.luteodontioides* due to its shorter capitate cystidia (11.5–16.5 × 3–4.5 µm) and its shorter basidia (10.5–16.5 × 2–4 µm; Guan et al. (2023)); *X.laurentianus* differs from *X.luteodontioides* due to its poroid to labyrinthiform hymenial surface and its wider basidia (18–26 × 4.5–5.5 µm) and its longer basidiospores (5–6 × 2.5–3.5 µm; Fernández-López et al. (2019)); *X.puerensis* differs from *X.luteodontioides* due to its poroid hymenial surface and its larger basidiospores (6–7 × 4.5–5.5 µm; Guan et al. (2023)); *X.subflaviporus* differs from *X.luteodontioides* due to its poroid hymenial surface and its shorter, wider basidia (8–18 × 4–5 μm; Chen et al. (2018)).

Morphologically, *X.poroides* resembles *X.daweishanensis*, *X.fissuratus*, *X.laceratus* and *X.wenshanensis* by the capitate cystidia. However, *X.daweishanensis* differs from *X.poroides* due to its odontioid hymenial surface and its shorter basidia (11–15.5 × 2.5–4 µm; Guan et al. (2023)); *X.fissuratus* differs from *X.poroides* due to its grandinioid hymenial surface; *X.laceratus* differs from *X.poroides* due to its grandinioid hymenial surface and its longer capitate cystidia (15.4–24.7 × 3.8–4.7 µm; Qu et al. (2022)); and *X.wenshanensis* differs from *X.poroides* due to its grandinioid hymenial surface and its shorter basidia (8–15.5 × 3–5 µm; Luo et al. (2022)).

Morphologically, *X.wumengshanensis* is similar to *X.asiaticus*, *X.laceratus*, *X.puerensis*, *X.punctus* and *X.wenshanensis* by having the ellipsoid basidiospores. However, *X.asiaticus* differs from *X.wumengshanensis* due to its hydnoid hymenial surface and its narrower basidiospores (4–5.2 × 2.8–3.5 µm; Zhang et al. (2024)); *X.laceratus* differs from *X.wumengshanensis* due to its shorter basidia (11–17.5 × 3.2–5.5 µm) and its narrower basidiospores (3.9–5.3 × 2.6–4.1 µm; Qu et al. (2022)); *X.puerensis* differs from *X.wumengshanensis* due to its poroid hymenial surface and its shorter basidia (14.5–20 × 5–7 µm; Guan et al. (2023)); *X.punctus* differs from *X.wumengshanensis* due to its smooth hymenial surface and its smaller basidiospores (2–4 × 1.5–2.5 µm; Luo et al. (2022)); and *X.wenshanensis* differs from *X.wumengshanensis* due to its smaller basidia (8–15.5 × 3–5 µm) and its smaller basidiospores (3–5 × 2–3.5 µm; Luo et al. (2022)).

Wood-inhabiting fungi, a unique group of Basidiomycota, have been identified through morphological, phylogenetic and cytological studies in China (Wu et al. 2020). Currently, forty-five species of *Xylodon* have been documented in China (Riebesehl and Langer 2017; Viner et al. 2018; Riebesehl et al. 2019; Shi et al. 2019; Luo et al. 2021a; Ji et al. 2022; Guan et al. 2023; Liu et al. 2023; Zhang et al. 2024; Zhao et al. 2024). However, the species diversity of *Xylodon* in China, particularly in the subtropical and tropical regions, remains largely unexplored. This paper contributes to our understanding of fungal diversity in these areas and underscores the urgent need for further fieldwork and molecular analyses to discover new taxa.

## Supplementary Material

XML Treatment for Xylodon cremeoparinaceus

XML Treatment for Xylodon luteodontioides

XML Treatment for Xylodon poroides

XML Treatment for Xylodon wumengshanensis

## ﻿Additional information

### Author ORCIDs

Qi Yuan https://orcid.org/0000-0002-6732-1656

Changlin Zhao https://orcid.org/0000-0002-8668-1075

### Data availability

All of the data that support the findings of this study are available in the main text.
